# A preliminary study of a lifetime long-term care costs estimation model based on changes in care level: implications for sustainable long-term care in Japan

**DOI:** 10.1186/s12877-026-07643-z

**Published:** 2026-05-19

**Authors:** Minoru Kumaoka, Takako Tsutsui, Kan Katori, Takashi Kinoshita

**Affiliations:** 1 Graduate School of Health and Welfare, Research and Development Center Saitama Prefectural University, Saitama, Japan; 2 Yasashii Te Co., Ltd., Tokyo, Japan; 3 Graduate School of Social Sciences, University of Hyogo, Kobe, Japan; 4 Graduate School of Human Science and Environment, University of Hyogo, Himeji, Japan

**Keywords:** Long-term care, Cost prediction, Care level changes, Japan, Aging society, Machine learning

## Abstract

**Background:**

Japan faces an unprecedented demographic shift, characterized by the world’s most rapidly aging population and a projected surge in annual deaths, leading to a “frequent death society.” This trend places substantial fiscal pressure on national healthcare and long-term care (LTC) systems, with expenditures already representing a significant share of gross domestic product (GDP) and continuing to rise. To support sustainability, accurate and proactive cost-prediction models are needed for resource allocation and policy planning. Japan’s Long-Term Care Insurance (LTCI) system, established in 2000, provides services based on a seven-level care needs certification, which directly determines monthly benefit limits and strongly influences overall LTC expenditures. Ongoing revisions to the certification system underscore the need to understand how changes in care levels relate to future costs. Traditional cost-prediction models often rely on static, short-term aggregates and may miss dynamic spending patterns. In contrast, data-driven approaches (e.g., trajectory-based methods and machine learning) can identify evolving patterns over longer periods and leverage routinely collected data to enable earlier risk stratification and targeted interventions.

**Objectives:**

This preliminary study addresses a specific research gap by uniquely focusing on estimating lifetime LTC costs based on "changes in care levels," utilizing only initial (first three months) service utilization data and associated costs, without requiring extensive patient background information. Although we refer to “lifetime cost estimation,” the present analysis is based on observed service utilization and expenditures over a 12–36-month observation window; therefore, findings should be interpreted as an estimation of longer-term cost trajectories rather than directly observed lifetime costs.

**Methods:**

We analyzed data from 5,925 LTC users who initiated services at one of 91 home care service centers operated nationwide by a single company in Japan in June 2015 or later, continued service use for 12–36 months, and were certified at Care Levels 1–4. The provider is a privately held (non-listed) corporation; therefore, publicly available audited financial statements and dividend policies are limited. As supplementary context, we referenced publicly available Official Gazette (Kanpo)-derived corporate information (Kanpo-derived database; CATR) [1]. The outcome was monthly average LTC service cost. Predictors included initial care level, first-month costs, binary indicators for seven LTC service types used in the first month, binary indicators for changes in costs for each service type during the first three months, and interruption of LTC service use during the first three months. We constructed prediction models using random forest and multivariable linear regression, with an 80/20 split for training/validation. For cost comparisons, users were categorized into a Maintenance/Improvement Group (final care level unchanged or improved from baseline) and a Deterioration Group (final care level worsened from baseline).

**Results:**

The Deterioration Group showed significantly higher costs from the first month, particularly among users with higher independence (Care Level 1 or 2), which may reflect early anticipation of deterioration by care providers. Predictive performance was high for both random forest (R2 = 0.677 in the preliminary study) and linear regression models. The linear regression model performed best primarily in the stable Care Level 1 Maintenance Group, whereas the random forest model performed better across most other cohorts, particularly at higher Care Levels (3 and 4). High predictive accuracy was achieved without requiring basic patient attributes (e.g., age, sex) or underlying disease information. In contrast, predictive performance was relatively low in the Care Level 1 Deterioration Group, suggesting greater heterogeneity in cost trajectories among users who are mild at baseline but subsequently deteriorate.

**Conclusions:**

This preliminary study demonstrates the feasibility of estimating longer-term LTC cost trajectories based on early service utilization patterns, highlighting the potential role of care managers in shaping future cost trajectories. These findings may inform efforts to enhance the fiscal sustainability and quality of Japan’s LTCI system.

## Introduction: demographic imperatives and fiscal pressures on Japan’s long-term care system

The world confronts an unprecedented demographic transformation, marked by an escalating aging population. Japan, in particular, stands as the most rapidly aging nation globally, transitioning into a "frequent death society" with a projected surge in annual mortality. In 2023, a substantial 79.1% of deaths in Japan occurred in individuals aged 75 years or older, with 52.0% in those aged 85 years or older, unequivocally demonstrating the combined effects of extended life expectancy and mortality concentration in advanced age groups [2]. This profound demographic shift exerts immense fiscal pressure on the national healthcare and long-term care (LTC) systems, as expenditures already consume a significant portion of the Gross Domestic Product (GDP) and continue to rise annually. For instance, in 2023, Japan's annual medical expenditures reached approximately ¥47.3 trillion (approximately USD 411 billion), and LTC expenditures amounted to about ¥11.2 trillion (approximately USD 97.4 billion), with both categories projected to increase further [3, 4].

To ensure the enduring sustainability of these vital social systems, the development of accurate, dynamic, and proactive cost prediction models is indispensable for effective resource allocation and informed policy formulation. Japan's Long-Term Care Insurance (LTCI) system, established in 2000, operates as a distinct social insurance scheme, providing comprehensive services structured around a seven-level care needs certification. These care levels directly dictate monthly benefit limits, thereby rendering the certification process a critical determinant of overall LTC expenditures. The ongoing "continuous review" of this certification system signifies a systemic response to the fundamental challenge of balancing increasing demand from an aging population with finite resources. The direct linkage between care levels and benefit limits implies that any alterations to certification criteria or assessment methodologies could have widespread fiscal repercussions across the entire system. This highlights a persistent policy effort to maintain a delicate equilibrium between providing appropriate care and ensuring financial viability, suggesting that the government actively seeks data-driven insights to inform these crucial policy adjustments [5].

Traditional healthcare cost prediction models have often relied on static, short-term aggregate values, which inherently overlook dynamic spending patterns and consequently exhibit limited predictive accuracy. However, the advent of novel data-driven approaches, such as group-based trajectory modeling and advanced machine learning techniques, offers superior predictive capabilities by identifying distinct, evolving spending patterns over extended periods [6–8]. These sophisticated methodologies enable the leveraging of routinely collected data to proactively classify patients and to target interventions with greater precision and efficacy.

This study specifically addresses a critical research gap. While the association between overall multimorbidity (the coexistence of multiple chronic diseases) and total medical and LTC expenditures has been a subject of increasing interest [9], its comprehensive combined impact remains largely unquantified globally. Recent Japanese research has begun to bridge this gap by demonstrating a clear association between multimorbidity and increased total healthcare costs. Furthermore, the conventional understanding of end-of-life spending as a sudden "surge" is being challenged by findings of "high persistent" cost patterns that extend years before death, thereby necessitating a shift towards chronic, long-term care planning [10]. The integration of behavioral indicators, such as medication adherence and "moral hazard," into predictive models like the Adherence Score for Healthcare Resource Outcome (ASHRO), also represents a crucial advancement, recognizing patient behavior as a powerful predictor of lifetime costs and health outcomes [11].

### Rationale for excluding clinical diagnosis variables

This preliminary study intentionally adopted a service-utilization–only approach and did not include clinical diagnosis variables (e.g., dementia, stroke, fractures). This design reflects practical settings where long-term care service records cannot be routinely linked with medical claims/clinical records due to data governance, availability, or privacy constraints. Our aim was to examine whether early service utilization patterns and costs during the first three months can provide sufficient predictive signals for subsequent lifetime care expenditures. If initial behavioral patterns (service utilization, changes in care levels) prove to be highly predictive, it becomes feasible to reduce data burden and accelerate model development and deployment. This implies a shift in focus from "who the patient is" (static profile) to "how the patient is progressing" within the care system (dynamic behavior). This holds profound implications for the practicality and scalability of predictive analytics in healthcare, particularly in systems like Japan's, where care managers play an active role in service planning. It suggests that the care management process itself generates valuable predictive signals.

## Preliminary study overview: design and analytical framework

This retrospective longitudinal study utilized data obtained with user consent from electronic care records across 91 home care service centers, operated nationwide in Japan by a single company. These centers are operated by a single nationwide for-profit corporation (Yasashii Te Co., Ltd.), which functions as an integrated delivery system providing both care management and a comprehensive suite of home-based long-term care services. The study utilized data on the initial care level, the care level at the end of the target period, the LTC services utilized, and their associated costs, for older individuals who continued to reside in their familiar communities while receiving LTC services for a period of three years. The analysis encompassed 5,925 LTC service users who initiated service use in June 2015 or later, had utilized services for 12 to 36 months, and were certified at Care Levels 1 to 4. The average exchange rate between Japanese Yen and US Dollars from 2015 to 2018 was 113.1 JPY per 1 US Dollar.

The primary outcome measure was the monthly average LTC service cost for the target users. Explanatory variables included the initial care level (treated as a continuous variable), LTC costs in the first month (treated as a continuous variable), seven types of LTC services utilized in the first month (Home-visit LTC services, Outpatient day LTC services, Home-visit nursing care services, Lending welfare instruments, Home-visit bathing service, Short-term stay at a care facility, Sanatorium-type medical care facilities for older adults requiring care) (all treated as binary variables), and changes in LTC costs for each type of LTC service during the first three months (all treated as binary variables), as well as interruption of LTC service use during the first three months.

For statistical analysis, users were stratified into two groups: a "maintenance and improvement group" and a "deterioration group," and costs for each group were analyzed. Comparisons of service utilization and costs between both groups were performed using IBM SPSS Statistics 29, employing the Kruskal–Wallis test and paired-sample t-tests. For model construction, the dataset was randomly partitioned into a training dataset (80%) and a validation dataset (20%). Random forest analysis was conducted using NTT DATA Mathematical Systems Alkano 1.1.1. Linear regression analysis was also performed using IBM SPSS Statistics 29 with a stepwise method. Model accuracy was evaluated based on R2 scores, Mean Absolute Percentage Error (MAPE), and Root Mean Square Error (RMSE).

The decision to employ both random forest and linear regression analyses, and to explicitly discuss their respective trade-offs (random forest for high accuracy, linear regression for interpretability), reflects a sophisticated understanding of the needs in applied research. Predictive models must not only forecast but also facilitate decision-making. Clinicians and policymakers are more likely to trust a model if they comprehend its underlying logic. For instance, if a model suggests a patient's costs will be high, care managers need to understand why (e.g., specific services, early signs of deterioration) to intervene effectively. Thus, this dual approach demonstrates a nuanced appreciation for applied research in health economics. Random forest provides "what" is predicted (high accuracy), while linear regression offers "how" (which variables exert linear influence, their direction, and magnitude). This enables both resource optimization through accurate prediction and informed policy and clinical intervention design through interpretability.

## Key findings and quantitative insights

LTC service users were stratified into "maintenance and improvement" and "deterioration" groups, and cost disparities were meticulously analyzed. Care Level 1 users of home-visit LTC services in the maintenance and improvement group had first-month costs of USD 245.3 and monthly average costs of USD 245.9. These figures were significantly lower than those observed in the deterioration group, which recorded USD 324.0 for the first month and monthly average of USD 436.9. Consistently, for users across other care levels, the expenditures for the deterioration group were notably higher from the first month onward. These initial cost patterns serve as strong indicators of future cost trajectories.

The observation that users in the deterioration group, despite possessing high levels of independence (Care Level 1 or 2), were provided with a substantial number of services from the very first month suggests that their deterioration was anticipated. This increased initial service provision for seemingly independent patients is a direct consequence of early risk assessment and proactive care planning by care managers. This may indicate an association where early professional judgment by care managers leads to increased initial service allocation for at-risk individuals, which then serves as a predictive signal for future deterioration and higher costs. This highlights the dynamic interplay between human expertise (care manager's foresight) and observed data patterns (initial service utilization).

Most common LTC service users, outpatient day LTC services, in the deterioration group at both Care Levels 1 and 2 had significantly higher first-month and monthly average costs than the maintenance and improvement group. Conversely, for users at Care Level 4, who typically require extensive LTC services, there was no significant difference in first-month costs for outpatient day LTC services between the deterioration and maintenance and improvement groups. However, the first-month costs for home-visit nursing care services were higher in the deterioration group. This suggests that for higher care levels, specific intensive services like home-visit nursing, rather than general outpatient day care, may serve as earlier indicators of deterioration (Tables 1, 2 and 3).

**Table 1:**
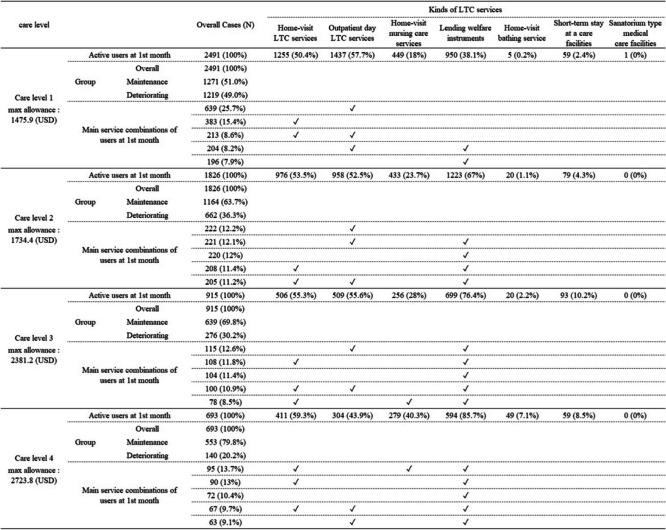
Main service combinations of users at 1st month

This table presents a summary of main service combinations for active users in the 1st month, categorized by care level and group (Overall, Maintenance, Deteriorating). The percentages in the "Main service combinations" rows indicate the proportion of users within each group who utilized that specific combination of services. Checkmarks (✓) denote the inclusion of a service in a combination. The percentages for individual service types (e.g., Home-visit LTC services) indicate the proportion of active users in the 1st month who utilized that service

**Table 2:**
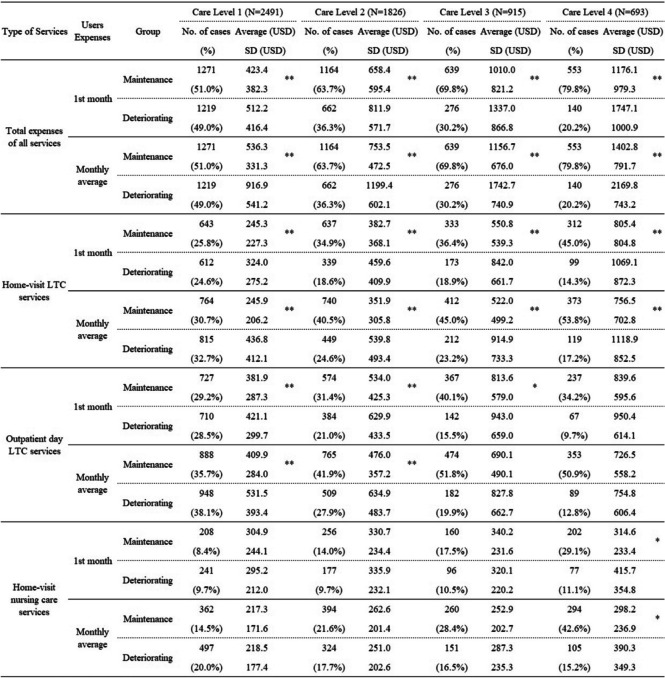
Comparison of user expenses in the first month and in the monthly average

This table presents a detailed comparison of user expenses in the first month and monthly average for various LTC services, stratified by care level and group (Maintenance vs. Deteriorating). All monetary values are in USD

^*^p<0.05

^**^p<0.01

**Table 3:**
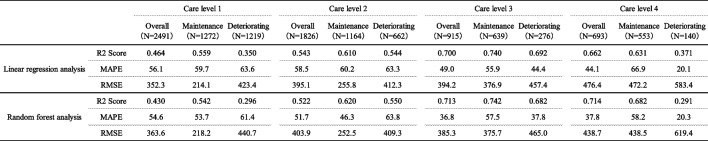
The prediction accuracy of each analysis for care levels

This table presents the prediction accuracy of linear regression and random forest analyses for different Care Levels (1-4) and user groups (Overall, Maintenance, Deteriorating). R^2^ Score, Mean Absolute Percentage Error (MAPE), and Root Mean Square Error (RMSE) are reported

## Multifaceted discussion: contextualizing the study’s contributions

### The predictive role of care managers

The high predictive accuracy of this model, particularly when based on initial service patterns, strongly suggests that care managers play a pivotal role in influencing future cost trajectories. Within the framework of the Japanese LTCI system, care managers act as the user's agent, not as resource allocators for the community. While the integration of care management and service provision within a single entity may theoretically introduce a "self-referral bias," empirical evidence in the Japanese LTCI system suggests that service combinations are predominantly dictated by users' physical and psychosocial status rather than agency characteristics [12]. Furthermore, research focusing on supplier density in Japan provides only limited evidence for systemic supplier-induced demand in the at-home care market [13]. Care managers, by arranging for users to receive the most needed services at the appropriate time, may be anticipating deterioration and optimizing LTC services according to care level. This proactive planning directly impacts initial service utilization and subsequent cost patterns.

The study posits that care managers act as Early Risk Assessors and Anticipatory Service Optimizers within the benefit limit, arranging for users to receive the services they need most at the appropriate time and that "those who plan the provision of care services (care managers) may be anticipating deterioration". Given that the model's high accuracy is predicated on "service utilization in the initial months", the "anticipatory adjustments" made by care managers are implicitly captured within the initial service utilization data. This implies that the model is not merely predicting based on raw patient data, but on data already shaped by expert human intervention. This elevates the role of care managers from mere service coordinators to active participants in shaping cost trajectories, with their decisions generating predictive signals. This suggests that enhancing the effectiveness of care managers (e.g., through better training, or data tools like this model) could directly lead to more efficient resource allocation and potentially a reduction in overall lifetime costs by optimizing early interventions [14]. It also suggests that part of the model's predictive power reflects the existing system's ability (human-driven) to identify and respond to early signs of deterioration.

This study is intended to complement, not replace, the situational judgment of care managers. Practitioner intuition and quantitative cost estimation operate at different epistemic levels and can serve different stakeholders. The following three use cases can be envisaged:

- *Policy and insurer-level planning*: Estimating lifetime cost burden for sustainability projections of the Long-Term Care Insurance system.
- *Municipal and facility-level resource allocation*: Informing capacity planning under the upcoming care-level distribution shifts.
- *Family counseling and informed consent*: Providing care managers and families with an evidence-based reference for anticipated care trajectories, which to date have largely been communicated through experience-based narratives.

A further consideration concerns the upstream role of the attending physician’s opinion report (Shujii Ikensho), a mandated document within Japan’s LTCI certification process. This report conveys the physician’s prognostic assessment and recommendations regarding the necessity of medical-type LTC services, most notably visiting nurse care, which is typically initiated based on physician recommendation, with periodic reports returned to the attending physician throughout the care episode. While our dataset—drawn from care service records of a single nationwide provider—did not include certification documents, the influence of the attending physician’s prognosis is implicitly reflected in the initial service mix observed for each user, particularly in the inclusion of visiting nurse services. The model can therefore be understood within a layered information flow: physician prognostic assessment (upstream) → care manager planning (midstream) → service utilization patterns (downstream, the input to our model). Although the upstream physician layer is not directly observed in this preliminary study, it exerts a structuring influence on the predictive signals captured in the initial service utilization data. The corresponding limitation, and a concrete plan for future research linking certification records (including the attending physician’s opinion report) with care service utilization data, is discussed in Sect. " Strengths and limitations of the preliminary study".

### LTCI system sustainability and policy implications

The high predictive accuracy achieved without requiring extensive patient background information provides timely empirical evidence directly relevant to the ongoing review of Japan's care needs certification system. The LTCI system incorporates "benefit limits" for each care level, which function as a monthly cap on service utilization. This significantly influences total costs based on the initial care plan. This systemic constraint further underscores the importance of accurate early prediction.

The finding that users certified at Care Level 1 but destined for deterioration exhibited significantly higher costs from the first month onwards suggests a critical gap between the static care level certification and the true, dynamic underlying care needs and risk. This indicates that the current LTCI assessment system may benefit from refinement in its early risk identification mechanisms, as care managers are already implicitly identifying and compensating for these "hidden high care needs" through increased service allocation.

The escalating costs of medical care impose significant fiscal burdens globally, making cost containment imperative. If this model can accurately estimate longer-term cost trajectories from initial signals, policymakers can adjust care needs certification criteria to identify individuals at risk of high lifetime costs earlier, guiding them toward appropriate and cost-effective interventions. It can also inform decisions regarding the setting of benefit limits at different care levels, ensuring they are fiscally sustainable while meeting patient needs. Furthermore, by designing reimbursement schemes and incentive structures that promote early interventions, as indicated by the model's predictive signals, policy can shift from merely capping costs to actively managing cost trajectories. This model provides a data-driven rationale for such a policy shift, enhancing the "fiscal sustainability and quality" of the LTCI system.

A critical consideration is the potential for unintended consequences if this model is widely promoted as a major expenditure prediction tool. There is a risk of Risk-Based Discrimination, where insurers or providers might be incentivized to ration services or unduly restrict care for individuals predicted to be high-cost, thereby compromising the LTCI's core mission of quality care. To mitigate this, the model must be strictly positioned as a tool for Early Intervention and Prevention Investment, serving as a trigger for focused re-assessment and proactive support—rather than a justification for cost restriction—to optimize long-term outcomes. Furthermore, mechanisms to ensure Model Transparency (Interpretability) are essential to prevent over-reliance on algorithms and preserve the professional discretion of care managers.

### Advancements in cost prediction methodology

This study's approach of predicting home care costs using random forest and linear regression, without relying on basic patient attributes (age, sex), underlying diseases, employment status, or community activities, represents a novel contribution. The utilization of data from a for-profit operator reflects a significant segment of Japan's diversified care landscape. Contrary to theoretical concerns regarding opportunistic behavior in for-profit sectors, previous studies have demonstrated that such entities maintain service quality and efficiency comparable to non-profit counterparts through competitive adaptation and human capital investment [15, 16]. Previous efforts have classified patients based on healthcare expenditure patterns, and factors such as treatment complexity, depression, medication adherence, smoking, chronic diseases, organ failure, neurodegenerative diseases, and fractures have been shown to influence costs. For instance, Mori et al. found that higher Charlson Comorbidity Index (CCI) scores were associated with higher annual total medical and LTC expenditures [17].

Teraoka et al. identified four distinct spending trajectories for LTC costs over five years before death (low persistent, late rise, progressive increase, high persistent), noting that persistently high medical spending was more common in men, while LTC constituted a high proportion of high persistent trajectories in women. This highlights the importance of longer observation periods and gender as a variable. Takura et al. developed ASHRO, an integrated predictive model for medical and LTC resource consumption based on health behaviors, using machine learning and big data. They found it accurately predicted future consumption and correlated with clinical outcomes. However, their model's sensitivity to LTC costs was reported to be low.

This study's findings on model performance demonstrate a nuanced trade-off between the two methods: while Linear Regression (LR) achieved superior predictive accuracy primarily for the stable, simpler cohort (Care Level 1 Maintenance Group), the Random Forest (RF) model generally achieved better or comparable accuracy across all other cohorts, demonstrating superior performance in the high-complexity Care Level 3 and 4 Overall Groups. This suggests that the complex, non-linear interactions inherent in higher care needs—such as the specific combinations of intensive services—are better captured by the RF algorithm. Conversely, the high interpretability of the LR model remains valuable for simple, stable care scenarios where costs are determined by more linear factors, such as the initial absolute service cost. The dual approach thus optimizes both the prediction accuracy (RF) needed for resource planning and the causal interpretability (LR) needed for policy design.

This study’s finding that it “achieved high accuracy in estimating care costs for older individuals without requiring any data on basic attributes such as age or sex, or information on underlying conditions, employment status, or community activities” contrasts with other research. For example, Teraoka et al. reported that “adding gender as an explanatory variable, extending the observation period, and setting the observation period to the end of life may improve predictive accuracy”. Mori et al. also stated that “higher CCI scores were associated with higher annual total medical and long-term care expenditures in older adults”. Furthermore, Pozo-Rubio et al. noted that age, sex, and educational level were factors in catastrophic LTC expenditure [18]. Importantly, this preliminary study intentionally adopted a “service-utilization–only” approach using routinely collected data from the initial three months, reflecting practical settings where clinical diagnoses and other background variables (e.g., comorbidity indices) may not be readily linkable/available across systems. This design choice raises the question of whether such factors are entirely unnecessary as direct inputs, or whether the core predictors in this study—early service utilization and costs—implicitly capture part of their effects through care managers’ initial care planning. At the same time, the exclusion of explicit clinical information may partly explain the lower predictive performance observed in heterogeneous subgroups such as the Care Level 1 deterioration group. Future research should therefore evaluate hybrid models that integrate early service-utilization signals with demographic and clinical/claims-based variables (including diagnoses and comorbidity measures) when linkage is feasible, particularly for longer-term (beyond 36 months) or end-of-life cost predictions as suggested by Teraoka et al.’s 5-year trajectory study.

### Contextualizing with broader determinants of LTC costs

Beyond clinical and demographic factors, socioeconomic status and social engagement have been shown to significantly influence healthcare and LTC expenditures. Hamada et al. found that among Japanese individuals aged 75 years or older, lower household income was associated with fewer physician visits but longer hospital stays and higher total medical and LTC expenditures [19]. This suggests that financial barriers can lead to delayed care, resulting in more severe conditions and higher costs. Similarly, Saito et al. demonstrated that employment status and frequent participation in community activities (e.g., hobbies, sports clubs, volunteering) were associated with significantly lower cumulative LTC costs and mortality rates over a 6-year period. For instance, individuals participating in hobbies or sports group activities at least twice a week had approximately 1.23 to 1.18 thousand USD lower costs per person over 6 years compared to non-participants. Employed persons also incurred about 0.55 to 0.64 thousand USD lower costs than retirees [20]. These findings underscore the importance of social determinants of health in influencing LTC expenditures and highlight potential avenues for cost reduction through public health interventions promoting active aging.

Furthermore, the concept of frailty and sarcopenia (PF&S) has emerged as a critical predictor of healthcare and LTC utilization. Sicsic et al. showed that PF&S indicators are strongly associated with increased use of formal and informal LTC services in community-dwelling older Europeans [21]. Notably, low-income frail older individuals faced a higher risk of hospitalization and emergency admissions, and exhibited higher LTC utilization, particularly for formal care services like meals on wheels, public transportation, and visiting nurses. This emphasizes the intersection of clinical vulnerability (frailty) and socioeconomic disadvantage in driving high healthcare and LTC costs, suggesting that interventions targeting frailty, especially in vulnerable populations, could yield significant cost savings.

These broader determinants of LTC costs, while not directly included as input variables in our preliminary model, provide crucial context for interpreting our findings. The predictive role of care managers, as identified in our study, may implicitly account for some of these factors, as care managers' decisions are likely influenced by their assessment of a patient's overall social and economic circumstances, in addition to their clinical needs. Future research should explore how these multifaceted determinants can be explicitly integrated into predictive models to enhance their comprehensiveness and inform more holistic policy interventions.

### Strengths and limitations of the preliminary study

This preliminary study possesses several significant strengths and limitations in its design and findings.

#### Strengths

- *High Predictive Accuracy and Efficiency*: The study achieved high predictive accuracy (R2 up to 0.714) using only initial (first three months) service utilization data, without requiring extensive patient background information. This characteristic enhances the model's practicality and efficiency.
- *Balance of Accuracy and Interpretability*: By employing both random forest and linear regression methods, the study strikes a balance between achieving high predictive accuracy and maintaining model interpretability.
- *Policy Relevance*: The study's focus on "changes in care levels" directly addresses a critical aspect of Japan's LTCI system.

#### Limitations

- *Limited Observation Period*: This study is preliminary, and its observation period was limited (service utilization period 12 months to 36 months). Teraoka et al.'s research suggests that end-of-life cost trajectories can span over five years or more. Because the observation period was limited to 12–36 months, “lifetime” cost estimates in this preliminary study should be interpreted as estimations of longer-term trajectories rather than directly observed lifetime expenditures.
- *Input Variables Scope*: While excluding basic attributes (age, sex) and underlying diseases as direct input variables is a strength in terms of data collection simplicity, it may limit the model's generalizability or its ability to capture all nuances of cost drivers, as suggested by other research. This limitation is particularly evident in the Care Level 1 Deterioration Group, which yielded the lowest R2 score. This suggests that the complex, heterogeneous factors driving deterioration in this mild-care but high-risk cohort (such as the onset of specific co-morbidities, socioeconomic decline, or frailty) are not fully captured by the initial, low-level service utilization data alone.
- *Absence of Behavioral Indicators*: This study did not explicitly consider behavioral indicators such as medication adherence or "moral hazard" as direct inputs. Takura et al.'s ASHRO model indicates the importance of these indicators in cost prediction.
- *Influence of hospital discharge*: Regarding the potential influence of hospital discharge, while intensive service utilization often characterizes the transition to home care, these "recovery trajectories" typically exhibit a subsequent decline in expenditures as functional stability is regained [10]. In contrast, the sustained or escalating cost patterns observed in our "deterioration group" suggest that these early expenditures capture a progressive need trajectory rather than a temporary post-discharge recovery spike. However, the lack of explicit hospital discharge markers remains a limitation for further granular analysis.
- *Limited Generalizability*: The study analyzed data from older individuals who continued to reside in their familiar communities while utilizing LTC services for three years, potentially excluding patients experiencing more rapid deterioration or facility institutionalization. This may limit the model's generalizability to the full spectrum of LTC users.
- *Absence of Explicit Clinical Diagnosis*: The absence of explicit clinical diagnosis variables may have particularly affected prediction in the Care Level 1 deterioration subgroup, where early utilization patterns may not fully capture emerging disease-related risks; this supports the need for future hybrid models when data linkage is possible.
- *Provider-side Incentives*: Provider-level financial indicators (e.g., dividend policy, profitability) and community-level market structure (e.g., relationships/competition with other providers) were not observable in our dataset; therefore, mechanisms such as supplier-induced demand cannot be empirically tested in this study. The provider is privately held and does not publicly disclose audited financial statements or dividend policies. As supplementary context, publicly available Kanpo-derived corporate information [1] shows positive net income in each fiscal year displayed (FY2018–FY2025; 55,092–164,863 thousand JPY) and an equity ratio of approximately 9.4–13.1%; however, these figures are insufficient to evaluate provider-side incentives.
- *Lack of End-User Validation*: The practical utility of the proposed estimation model has not yet been validated with care managers or other end-users in the field. Future research should incorporate structured feedback from care professionals to assess the model's usefulness in real-world planning and counseling contexts.
- *Absence of Doctor’s Opinion Report Data*: Japan’s LTCI certification process incorporates the attending physician’s opinion report (Shujii Ikensho), which conveys prognostic information and recommendations regarding the necessity of medical-type LTC services such as visiting nurse care. Our dataset, drawn from care service records of a single nationwide provider, did not include these certification documents. Although the physician’s prognostic influence is implicitly captured in the initial service mix observed in our data—particularly in the inclusion of visiting nurse services—future research that directly integrates physician opinion reports with care service utilization data would enable more precise modeling of medical-type service trajectories and their associated cost implications, particularly for users with complex multimorbidity or higher medical care needs. Such hybrid modeling represents a natural extension of the present preliminary study.

The study's target population is defined as "older individuals who continued to reside in their familiar communities while utilizing LTC services for three years" and who "had utilized services for 12 to 36 months and were certified at Care Levels 1 to 4". This implies a focus on a specific subset of LTC users. Real-world LTC users are highly diverse, and rapid deterioration, early institutionalization, or end-of-life care often involve different cost patterns and service needs. The study's focus on "community-dwelling" and "12–36 months" may exclude the most complex and costly cases. The remarkable accuracy of this model might be specific to a relatively stable, community-dwelling population. Future research should validate the model's robustness and generalizability across the entire spectrum of LTC users, including those with more severe conditions or in different care settings, to confirm its broader applicability. This is essential for a model intended to inform system-wide policy.

## Future research and policy recommendations

### Recommendations for enhancing model accuracy and robustness

Current models demonstrate excellent performance with minimal data, but future research should consider incorporating variables identified as influential in other studies. These include:

- *Sex*: Teraoka et al. highlight the influence of sex on persistently high medical versus LTC expenditures.
- *Socioeconomic Status*: Identified as a factor influencing economic hardship and LTC costs.
- *Underlying Diseases and Multimorbidity Patterns (e.g., CCI scores)*: Mori et al. found these to be strong drivers of total medical and LTC costs.
- *Behavioral Indicators (e.g., medication adherence, "moral hazard")*: Takura et al.'s ASHRO model found these relevant for cost prediction.

Extending the observation period to the end of life (beyond 36 months), as suggested by Teraoka et al., could significantly improve predictive accuracy and provide a more complete picture of longer-term (potentially lifetime) cost trajectories. Furthermore, a detailed examination of the linear regression model could reveal which specific LTC services have the greatest impact on costs, providing actionable insights for care managers and policymakers. Exploring hybrid models that combine the strengths of the current model (initial care level changes) with traditional demographic and clinical variables could potentially achieve even higher predictive accuracy and broader applicability.

### Practical applications in clinical settings

This model can serve as a valuable tool for care managers to proactively identify patients at risk of escalating costs, enabling early and targeted interventions. It could also assist hospital dental clinics in efficiently triaging dental services by providing insights into the overall care needs and potential cost trajectories of older patients with complex multimorbidity. The model can support Advance Care Planning (ACP) discussions by offering data-driven insights into future cost trajectories, helping patients and their families make informed decisions.

### Policy considerations for strengthening the LTCI system

The insights derived from this model can directly inform the ongoing review of Japan's care needs certification system, enabling data-driven adjustments to benefit limits and service allocation. Considering macro-level support mechanisms, such as increased reimbursement for dental treatment of complex multimorbidity patients under the public insurance system, is crucial to incentivize appropriate care for high-needs individuals [22]. Furthermore, fostering collaboration between hospitals and primary care facilities, and among various healthcare professionals (dentists, physicians, nurses, dental hygienists), as highlighted in 17, can ensure integrated and efficient care for multimorbid older individuals.

## Conclusion

This preliminary study makes a significant contribution by demonstrating the feasibility of estimating longer-term (potentially lifetime) LTC cost trajectories in Japan based on initial care level changes and early service utilization data, without requiring extensive patient background information. Its high predictive accuracy and insights into the predictive role of care managers offer a novel approach to cost prediction.

The findings of this study hold profound implications for improving care delivery and managing complex multimorbidity in Japan's rapidly aging society. By enabling the proactive identification of cost trajectories, this model serves as a powerful tool for enhancing the fiscal sustainability and quality of the LTCI system. While preliminary, this research lays a robust foundation for future, more comprehensive studies aimed at further refining predictive models, integrating diverse data sources, and ultimately informing evidence-based policies to ensure equitable and sustainable long-term care for the global aging population.

## Data Availability

The datasets generated and/or analyzed during the current study are not publicly available due to ethical restrictions and regulations set by the data providers (e.g., Federation of National Health Insurance Associations). However, data are available from the corresponding author for researchers who meet the criteria for access to these confidential data. Requests to access the data should be submitted to the corresponding author.
